# 

**Published:** 1987-02

**Authors:** 


**Contents**

5 **Editorial**

6 **Should every cow carry a government health warning?**

J. R. A. Mitchell

9 **Bogus Allergy treatment**

T. J. David

12 **Redevelopment of Frenchay Hospital**

John S. M. Zorab

14 **Changing face of Ham Green Hospital**

Dennis W. Wright

15 **From our Foreign Correspondents**

17 **Surgical Club of the South West**

Meeting at Plymouth

21 **Woodcarving**

Alec S. Baxter

26 **Obituary**

Professor John Clutton Brock

Mr Anthony Palin

